# Using Sentinel-1 satellite imagery to quantify oil palm cultivation: A case study from Cameroon

**DOI:** 10.1371/journal.pone.0337475

**Published:** 2025-12-31

**Authors:** Christopher Chalmers, Elizabeth D. Crook, Ada N. Acobta, Kyle Manley, Benis Egoh

**Affiliations:** 1 Department of Earth System Science, University of California, Irvine, Irvine, California, United States of America; 2 Cooperative Institute for Research in Environmental Sciences, University of Colorado, Boulder, Boulder, Colorado, United States of America; World Bank Group, UNITED STATES OF AMERICA

## Abstract

Tropical deforestation is increasing globally, putting forest ecosystems and the ecosystem services they provide at risk. The widespread use of palm oil in fast food, cosmetics, household cleaners, and other products has led to a high demand for oil palm trees, *Elaeis guineensis*, in tropical regions due to their favorable growth conditions and low production costs. Thus, rapid agricultural expansion poses a significant threat to regions reliant on palm expansion for economic growth, such as the African tropical nation of Cameroon. This study aims to quantify the change in oil palm extent from 2015 to 2025 in Cameroon, using Sentinel-1 SAR satellite data and the machine learning algorithm Random Forest. Our findings reveal an increase in oil palm cultivation ranging from 13-55% in the focus areas, with the South Region showing the greatest change in forest cover as a result of oil palm expansion. We also discuss the use of Sentinel-1 satellite data and machine learning for similar studies monitoring oil palm deforestation in tropical regions.

## Introduction

Tropical rainforests are biodiversity hotspots [1,2] that provide critical ecosystem services such as food provision, raw materials (i.e. timber), carbon sequestration, air and water purification, and recreation/tourism [3–6]. Tropical rainforests are especially valuable for the global carbon budget as they store approximately 195 petagrams of carbon (β25% of global carbon), and are therefore effective sinks for atmospheric carbon, mitigating climate change [3,7–9]. Tropical rainforests are under threat largely due to agricultural and urban expansion focused on increasing urban development [10,11]. Understanding and monitoring agricultural-driven deforestation in tropical regions is critical to assessing and mitigating the impacts to ecosystem services [12].

Rapid deforestation is occurring in tropical forests throughout Southeast Asia, Latin America, and Africa, in part due to oil palm *(Elaeis guineensis)* cultivation [6,13–15]. Oil palm cultivation is expanding rapidly due to high production yields and low costs relative to other vegetable oils, enabling its widespread use in biofuels, fast foods, cosmetic products, and household cleaners [6,13–17]. In fact, oil palm is now the most commonly consumed vegetable oil source globally and accounts for up to 45% of agricultural deforestation [13]. Efforts to understand the impact of oil palm on forest loss are mainly concentrated in Southeast Asia, while the expansion of the oil palm industry in African nations remains largely unexplored [15]. The Congo Basin Rainforest is a suitable oil palm growth region that contains up to 30% of the world’s tropical forest carbon stock [15]. Little is known about the rate at which oil palm plantations are replacing tropical forests in Africa, and particularly in Cameroon which currently has one of the highest deforestation rates of any country [18,19]. Local socio-political factors within Cameroon, such as the Anglophone crisis, influence land use decisions and introduce additional sustainable agriculture challenges [20]. It is therefore difficult to assess the impact of the oil palm industry on biodiversity and valuable ecosystem services, and Cameroon would benefit from accessible methods to track palm oil cultivation over time.

Oil palm concessions in Cameroon have been located in previous studies using satellite imagery from Landsat-5 TM, Landsat-7 ETM+, Landsat-8, Sentinel-1, Sentinel-2, QuickBird, and ALOS-PALSAR [21–24]. Confirming tree locations also requires ground-truth data, either from field observations or visual interpretation on Google Earth imagery. Current research in Cameroon employs multispectral satellites, a combination of multispectral and synthetic aperture radar (SAR) satellites, and occasionally field data to enhance image quality and thereby spatially highlight oil palm [14,15,25–27]. To our knowledge, limited studies have attempted to detect oil palm plantations in Cameroon using only Sentinel-1 observations. Because it is an active satellite, Sentinel-1 satellite imagery can penetrate various layers of the Earth’s atmosphere, including clouds, which are nearly constant in tropical regions like Cameroon [28–30]. Additionally, Sentinel-1 data is easily accessible with a return period of six days, preventing the need for constant on-ground monitoring. A combination of optical and SAR satellites to detect palm have been used in several studies, but more recent research has shown that this combination may not be required [31,32]. Sentinel-1 data may therefore detect oil palm effectively on its own.

Here, we apply a Random Forest (RF) classification algorithm to Sentinel-1 SAR imagery to estimate oil palm extent and change from 2015 to 2025 across the Southwest and Littoral Regions near Buea, and the South Region near Kribi, Cameroon [31,32]. We use SAR data because of its capacity to penetrate cloud cover and adverse weather conditions that often limit optical imagery [31–33]. Additionally, SAR has been demonstrated to effectively detect oil palm cover in humid tropical environments [34]. Here, we use a streamlined and data-driven approach to detect oil palm extent using RF, integrating other approaches used in the field [31,32,35]. Specifically, we develop a RF model in ArcGIS Pro using Sentinel-1 VV and VH polarization band combinations to classify image composites from 2015 and 2025. This approach is designed to support efficient monitoring of oil palm dynamics and to contextualize ecosystem service risks associated with oil palm cultivation within established concession areas.

## Materials and methods

### Study area description

**Fig 1:**
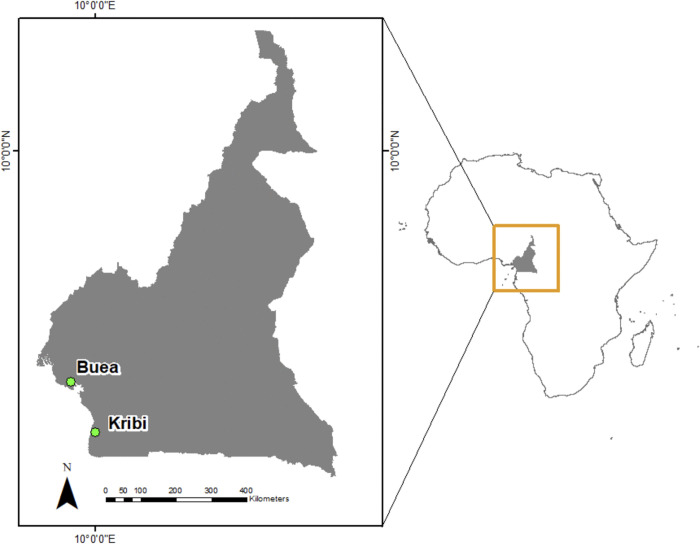
Study area. Map of Cameroon showing the two study areas: the Southwest and Littoral Regions (including Buea) and the South Region (including Kribi). Administrative boundary shapefiles were obtained from openAFRICA, a freely available database of geospatial data for Africa [39].

Cameroon, located in the Congo Basin rainforest, is a rich biodiversity hotspot, hosting approximately 850 species of birds and mammals and around 8,000 plant species [36]. The South and Southwest regions experience ideal conditions for oil palm cultivation, with annual temperatures averaging 20-25βC, monthly rainfall reaching up to 400 mm, and elevations around 470 meters above sea level [37]. The Southwest and South Regions of Cameroon, including the cities of Buea and Kribi (Fig 1), have been a vital region for oil palm cultivation since the early 1900s. Since Cameroon’s location within Africa’s tropical belt provides optimal conditions for oil palm growth, there has been significant economic development in the region [38]. Oil palm trees in the region are cultivated by three types of plantations: agro-industrial (typically over 1,000 hectares), smallholder (generally 1-50 hectares), and independent artisanal sectors (usually less than 10 hectares) [38]. These plantations often replace tropical forests and, less commonly, other tree crops. Smallholder plantations have rapidly expanded since the late 1990s, during which they planted 90,000 hectares of oil palm [38]. The continued growth of oil palm cultivation in Southwest Cameroon in the 21st century has also contributed to deforestation in the area [15].

### Data preparation

We obtained preprocessed C-band Sentinel-1 Level-1 Ground Range Detected (GRD) SAR imagery projected to Douala UTM Zone 32N from the Copernicus Data Space Ecosystem Browser, a European Space Agency (ESA)-supported web service. Sentinel data from this web service is provided on a free, full, and open basis. We use the Interferometric Wide Swath (IW) 10m x 10m Polarization acquisition mode, as it has been shown previously to be effective in monitoring forest change [31,32,35,40]. We focus on government-designated land-grant areas (concessions) allocated for oil palm cultivation in the Southwest/Littoral and South regions, both located in Cameroon. Choosing two distinct regions allows us to more rigorously evaluate the RF model’s performance in mapping oil palm extent across areas with documented cultivation.

To correct for terrain-induced geometric distortions, rasters were orthorectified using a Copernicus 30m digital elevation model. A Lee 7x7 speckle filter was applied to each raster to reduce granular noise caused by wave interference [31,41,42]. This approach greatly reduces speckle, which is apparent in single-date SAR imagery. We obtain five Sentinel-1 images per region and year, corresponding to monthly acquisitions from April through August. This yields 18 preprocessed images for analysis (Southwest/Littoral 2015, Southwest/Littoral 2025, South 2015, South 2025) and 5 images for training (Southwest/Littoral 2020), resulting in a total of 23 images used in the RF classification workflow (Table in S1 Table). Note that May 2015 did not have imagery available on Copernicus Browser, so only four out of five images were used for the Southwest/Littoral and South Regions for 2015.

**Fig 2:**
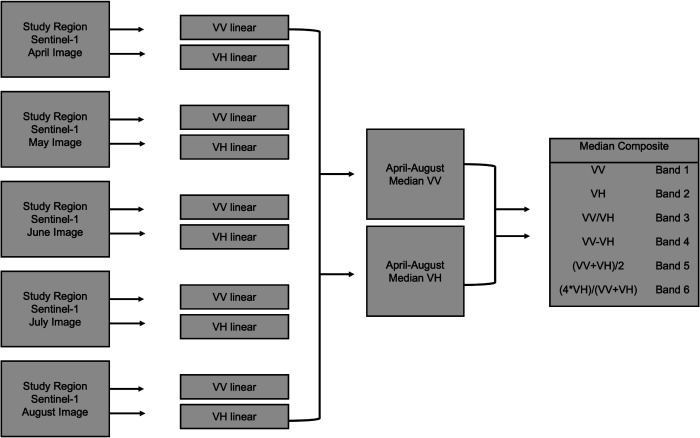
Methods workflow. Preprocessed, orthorectified, and speckle-filtered VV and VH bands from images acquired between April and August of 2015 and 2025 were extracted, and their median values were used to generate six-band composites for each region and year.

VV and VH polarization bands in linear units were extracted for the Southwest/Littoral and South Regions (Fig 2). We take the median of all five VV and VH bands from April to August for each respective year to represent multi-temporal conditions (Fig 2). In ArcGIS Pro, we created four six-band composites from these median bands, one composite per study region and year. Specifically, band 1 is *VV*, band 2 is *VH*, band 3 is VV/VH [31,32], band 4 is *VV*–*VH* [34,35], band 5 is β  +  ≈ [35], and band 6 is (4 * VH)/(VV  +  VH) [35] otherwise known as the radar vegetation index. VH polarization is particularly valuable for oil palm monitoring because it can detect objects smaller than the satellite’s 5.55 cm wavelength, such as the dense, fine folioles of oil palm which produces distinctive horizontal scattering signatures [32,43]. The Southwest/Littoral study area covers 5,122,725 pixels (1,553 km^2^) at a projected resolution of 17.4 meters, and the South study area covers 5,147,252 pixels (766 km^2^) at a projected resolution of 12.2 meters. These resolutions were automatically generated by Copernicus Browser, which automatically adjusts pixel size based on the extent of the selected area of interest.

### Description of the random forest model

RF is a well-known machine learning algorithm that has shown previous efficacy in detecting land cover characteristics [44–47], including oil palm cover [34,35]. RF is a decision tree-based machine learning algorithm that is commonly used in ecology due to its computational efficiency when handling big data, the mitigation of overfitting to training data, and the high precision of classification [34,44,48]. For this reason, we use RF to automate the classification of oil palm in satellite images. A detailed description of the algorithm is described in [44].

To train the RF model, we use the Train Random Trees Classifier tool in ArcGIS Pro. We digitize our classification variables on Google Earth Imagery in the Southwest/Littoral Regions from April 2020. Specifically, we digitize 464 oil palm polygons (75.2 km^2^), 161 other vegetation polygons (47.5 km^2^), 150 bare ground polygons (1.0 km^2^), 123 non-palm agriculture polygons (36.9 km^2^), 114 water polygons (46.3 km^2^), and 134 urban area polygons (4.7 km^2^). We ultimately train the model on a Sentinel-1 six-band composite image from 2020. A multitude of model hyperparameters are tuned to optimize the model (i.e. mtry, nodesize, ntrees). Once trained and internally validated, we use the RF model to classify our Sentinel-1 composite rasters from 2015 and 2025. The classification provides classed images for both areas of interest. To reduce remaining granular noise in the initial classifications, a region group analysis was performed to sieve and remove contiguous pixel groups smaller than a 1-hectare minimum mapping unit.

### Validation and area estimation

Validation was performed in two steps. First, an Out-of-Bag (OOB) internal validation was performed automatically by the Train Random Trees Classifier tool during model training. For each tree, roughly two-thirds of the training data were bootstrapped to build the model, while the remaining third served as OOB samples. Misclassifications by trees were used to compute the OOB error. Accuracy was then derived as 100% minus the OOB error percentage (88.1%).

Because the model was later applied to classify years other than 2020, we performed an external validation following [49] to ensure robustness. Given the study’s focus on explicitly mapping and characterizing oil palm extent, each raster was reclassified to a binary variable representing oil palm (1) or non-palm (0) for our accuracy assessments and results. For each year and region, we generated 400 random accuracy assessment points using an equalized stratified random sampling approach [49] to avoid over-representing either class. Ground-truth labels were assigned through visual interpretation of Google Earth imagery. A point was labeled as 1 (oil palm) if any palm canopy, frond, or structure was visible within or immediately surrounding the assessment point; otherwise, it was labeled as 0 (non-palm). When land cover was ambiguous, classification decisions were guided by the broader landscape context. Points were excluded only when reliable reference data was not possible, specifically when Google Earth imagery was too coarse, blurry, or cloud-covered for verification. In the South Region, we removed 18 palm and 18 non-palm points from the 2015 dataset, followed by 17 palm and 33 non-palm points from the 2025 dataset. Finally, we compared classified and ground-truth values using a confusion matrix to estimate user’s and producer’s accuracies for oil palm and non-palm. This external validation approach was selected to align with our goal of capturing the general extent of oil palm, including heterogeneous smallholder farms or native trees. Metrics derived from the resulting confusion matrices were also used to adjust palm area estimates with 95% confidence intervals [49].

## Results

**Fig 3:**
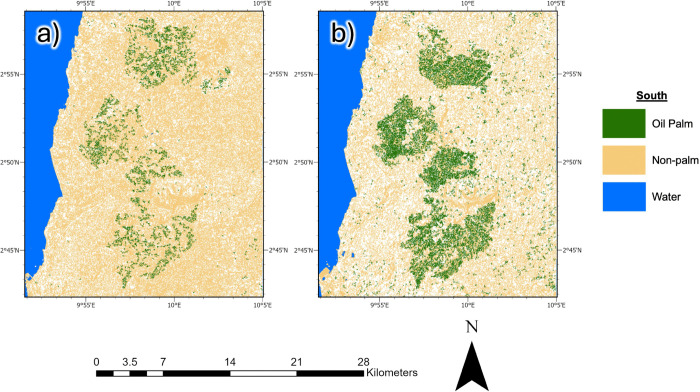
Southwest/Littoral Regions’ results. Oil palm and non-palm extent in the Southwest/Littoral regions in a) 2015, and b) 2025. The dark green in the map depicts oil palm, and beige represents a combination of other broadleaf forest species and non-oil palm vegetation, bare land and agriculture, water, and urban areas. Contains modified Copernicus Sentinel data (2015 and 2025).

Our RF model estimated the Southwest/Littoral study area as having an oil palm extent of 253.5 β 40.9 km^2^ in 2015, with the remaining 824.9±42.6 km^2^ identified as non-palm area (Fig 3A). By 2025, the oil palm extent increased to 284±40.9 km^2^, while the non-palm extent decreased to 710.7 ± 40.9 km^2^ (Fig 3B). This corresponds to a 12% increase in total oil palm extent and a concurrent 13.8% decrease in non-palm area over the 10-year study period. The model demonstrated robust performance across both years in the Southwest/Littoral Regions. For the palm class, the user’s/producer’s accuracies were 80.5%/86.6% in 2015 and 84.0%/84.8% in 2025, respectively. For the non-palm class, they were 87.5%/81.8% in 2015 and 85.0%/84.2% in 2025. Within the Southwest/Littoral study area, both distinct regions show divergent behavior. Littoral showed a clear expansion of detected oil palm between 2015 and 2025, with a net increase of 40.4 km^2^ (+44.91%). By contrast, Southwest contracted, with a net decrease of 9.0 km^2^ (–30.59%), indicating differing oil palm dynamics between the two regions (S1 Fig).

**Fig 4:**
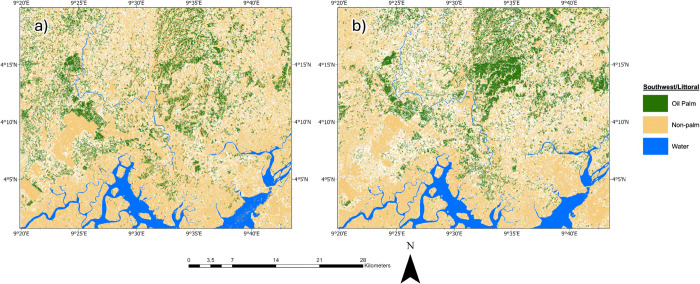
South Region’s results. Oil palm and non-palm extent in the South region in a) 2015, and b) 2025. The dark green in the map depicts oil palm, and beige represents a combination of other broadleaf forest species and non-oil palm vegetation, bare land and agriculture, water, and urban areas. Contains modified Copernicus Sentinel data (2015 and 2025).

Our RF model estimated the South study area as having an oil palm extent of 67.4 ± 19.2 km^2^ in 2015, with the remaining 457.1 ± 19.2 km^2^ identified as non-palm area (Fig 4A). By 2025, the oil palm extent increased to 105 ± 15.2 km^2^, while the non-palm extent decreased to 326.3 ± 15.2 km^2^ (Fig 4B). This corresponds to a 55.8% increase in total oil palm extent and a concurrent 28.6% decrease in non-palm area over the 10-year study period. The model also demonstrated robust performance across both years in the South Region. For the palm class, the user’s/producer’s accuracies were 97.8%/92.7% in 2015 and 95.2%/90.3% in 2025, respectively. For the non-palm class, they were 92.3%/97.7% in 2015 and 90.7%/95.4% in 2025.

## Discussion and conclusions

Using Sentinel-1 data to train a RF model, we detected a 13% and 55% increase in oil palm cover over a ten-year period in the Southwest/Littoral and South regions, respectively. This expansion coincided with decreases in other land cover classes, including broadleaf forest, non-oil palm vegetation, bare land and agriculture, water, and urban areas, by 13.8% in the Southwest/Littoral Region and 28.6% in the South Region. Therefore, these findings suggest a substantial growth of oil palm within the Southwest Cameroon region, combined with an overall loss of other land cover. The observed increase in oil palm cover was, on average, up to 2.4 times higher than the approximate 23% increase reported by the Food and Agriculture Organization for Cameroon between 2015 and 2021 [50,51]. However, it is important to note that our study areas were selected based on the likelihood of oil palm presence and therefore do not represent national-scale statistics or averages. Therefore, while the FAO’s measurements represent nationwide data, any additional discrepancy may be attributed to increased investment by international stakeholders, expansion by smallholder farmers, or differences between self-reported national statistics and satellite-derived measurements. If deforestation continues at this rate, critical losses to biodiversity and ecosystem services may result in the Southwest region of Cameroon.

Our Southwest/Littoral study area is divided by two of the ten regions of Cameroon, the Southwest Region and the Littoral Region. Here we identify divergent patterns in oil palm change from 2015 to 2025, with clear increases in the Littoral region, but in contrast to the patterns typically found across Cameroon [15,50], decreases in the Southwest region. That pattern aligns with the Anglophone crisis, which has been occurring within the Northwest and Southwest regions of Cameroon since 2016. During the crisis, agriculture and agro-industry were severely disrupted in the affected regions, with palm-oil output falling by approximately 40%, many palm-oil estates being abandoned, and thousands of jobs lost in the agricultural sector [52]. These crisis mechanisms explain why, even as Cameroon’s oil-palm area has generally expanded nationally, our change-detection isolates a conflict-zone contraction that national aggregates mask. In line with emerging remote-sensing work that quantifies conflict-induced damage to perennial crops at high resolution [53], our approach can also offer a practical path for crisis-aware monitoring of agriculture and local food-system risk [54].

Although the drivers of oil palm expansion are complex and nuanced [55], the concurrent loss in forest cover will have adverse impacts on ecosystem services. Thus, any benefits of replacing forest ecosystems with oil palm plantations will be subsidized by the public, who benefit and rely upon the ecosystem services provided by local forests [56]. For example, forest loss has implications on climate regulation and carbon sequestration ecosystem services provided by natural forests, which have been shown to hold 6-31% more carbon than oil palm plantations [57], providing global benefits. Conversion of natural forest to agricultural land destroys essential habitat for species, which has implications on the rich biodiversity of the area and is already materializing as environmental corridors to several protected areas in Cameroon are reportedly threatened [58]. Beyond carbon and habitat, benefits like resource provisioning and cultural services that communities across Cameroon rely upon for well-being, risk depletion [59,60]. For example, impacts on regional forest ecosystem intactness impacts food security [61] and recreational/tourism ecosystem services, like birding ecotourism [62] and other biodiversity-based tourism [63], which offer alternatives beyond agriculture for job-creation and poverty alleviation while preserving ecosystems and their services [64,65].

Results from our study are comparable to those observed in other tropical areas and may further serve to infer the relative age of tree stands over time. Specifically, the VV/VH ratio has been identified as a strong indicator of oil palm plantations and may also provide insights into plantation age within Cameroon [31]. For instance, in Gabon, directly to the south of Cameroon, the VV/VH ratio over palm plantations was approximately 4 during early development stages and increased to about 6-7 over a six-year period [31], suggesting a positive relationship between VV/VH ratio and palm maturity. In our study area, mean VV/VH values decreased from 6.23 to 6.18 in the South Region and from 5.34 to 5.23 in the Southwest/Littoral Region between 2015 and 2025. These patterns may indicate relatively older palms in the South Region and younger palms in the Southwest/Littoral Region, while also potentially reflecting recent planting activity. These numbers align with results in Gabon [31], though it warrants further investigation. Additional analysis and ground-truthing with datasets independent of Google Earth imagery are needed to confirm whether this approach can reliably estimate tree stand age in Cameroon and be generalized to other tropical regions in Africa.

Our oil palm estimates may additionally be limited by elevation, as topography can affect backscattering coefficients. For instance, Buea (within the Southwest Region) resides at a relatively high elevation, averaging approximately 1,000 meters above mean sea level on the southeastern slopes of Mount Cameroon, which overlaps with part of our study area [66]. Additionally, speckle or granular noise may also remain despite the use of the Lee 7x7 filter. Importantly, the presence of tree species with leaf structures similar in size to oil palm, such as acacias [32], coconut and sago palms [67], and other background forest species [31], can contribute to misclassification. While Sentinel-1 is effective at detecting oil palm, some stands of acacia, coconut palms, sago palms, and other similar forest vegetation may exhibit comparable backscatter signatures, necessitating careful consideration to avoid misclassification. Further research is warranted to optimize SAR band combinations for improved discrimination between oil palm and other surrounding vegetation, potentially through time-series analyses of growth stages using the VV/VH ratio [31] and the integration of optical data from sensors such as Sentinel-2 [31,32] as stacked techniques can show comparable accuracy [33]. Lastly, while Google Earth imagery was necessary for validation in this study, it is inherently limited by temporal mismatches, resolution constraints, and interpreter subjectivity, particularly in distinguishing oil palm from other palm species in smallholder or isolated contexts. Future assessments would be strengthened by validation using locally monitored ground-truth data [32], rather than relying on Google Earth imagery, to improve accuracy in Cameroon.

Sentinel-1 is particularly effective for oil palm detection in tropical regions due to its high spatial resolution and ability to penetrate cloud cover, which is a limitation that affects optical remote sensing data [30]. Therefore, our streamlined methods using Sentinel-1 data from the Copernicus Browser, along with our findings, offer valuable insights and a straightforward approach to assessing the rates of oil palm expansion within the tropical regions of Cameroon. The expansion of Cameroon’s oil palm industry is contributing to significant deforestation and the subsequent decline in biodiversity, impacting climate change both regionally and globally, and impacting the plethora of other ecosystem services locals rely upon for their livelihoods and well-being [12]. Thus, it is essential we continue to monitor oil palm expansion, deforestation, and the resulting impacts; using and refining our approach can help streamline these efforts, especially in response to the European policy on deforestation-free commodities [68], and should be explored further.

## Supporting information

S1 File Supporting figure and table. Supporting information PDF containing: (a) an oil palm change map showing areas of decreased oil palm extent in the Southwest Region (brown) and increased oil palm extent in the Littoral Region (green); and (b) a table listing Sentinel-1 images used in the analysis, obtained from the Copernicus Browser.
(PDF)

S2 File Accuracy assessment points. Accuracy assessment validation points (“Validation_Points.zip”), containing 400 validation points per region and year with the classified and referenced values.
(ZIP)
