# Supplementary material for: Using Sentinel-1 satellite imagery to quantify oil palm cultivation: A case study from Cameroon
Source: PLoS One. 2025 Dec 31;20(12):e0337475. doi: 10.1371/journal.pone.0337475 (PMC12755778; doi:10.1371/journal.pone.0337475)
Supplement: S1 File — Supporting information PDF containing: (a) an oil palm change map showing areas of decreased oil palm extent in the Southwest Region (brown) and increased oil palm extent in the Littoral Region (green); and (b) a table listing Sentinel-1 images used in the analysis, obtained from the Copernicus Browser. (PDF) [file pone.0337475.s001.pdf]

Supporting Information

Using Sentinel-1 Satellite Imagery to Quantify Oil Palm Cultivation: A Case Study from Cameroon

Christopher Chalmers[1], Elizabeth D. Crook[1], Ada N. Acobta[1], Kyle Manley[1,2], Benis Egoh[1]

1 Department of Earth System Science, University of California, Irvine, Irvine, CA, United States
2 Cooperative Institute for Research in Environmental Sciences, University of Colorado, Boulder, Boulder, CO, United States

1. Supporting Figures

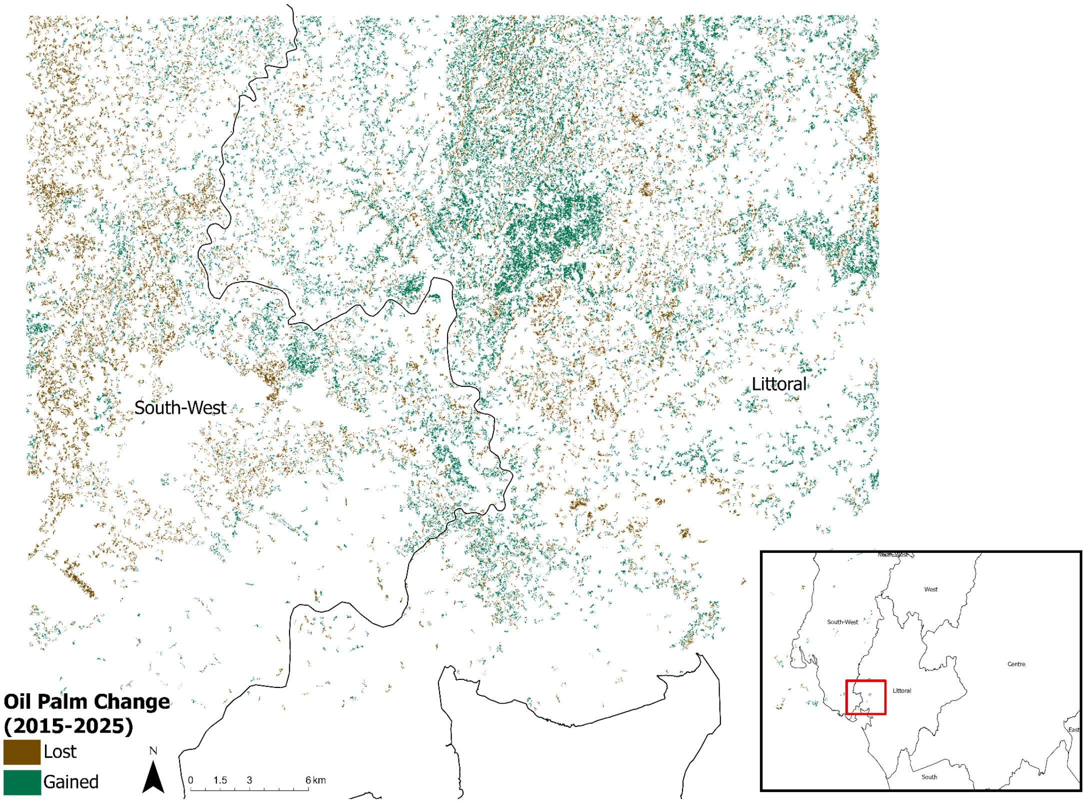

S1 Fig. Oil palm change map showing where oil palm extent has decreased in the Southwest Region in brown, and where oil palm extent has increased in the Littoral region in green. Contains modified Copernicus Sentinel data (2015 and 2025).

2. Supporting Tables

S1 Table. Sentinel-1 images used in the analysis, obtained from the Copernicus Browser. Note that on some days, multiple products are merged to create the singular images that we downloaded. In total, 18 preprocessed images (from 25 products) were used for analysis, and 5 images (from 10 products) were used for model training.

Area	Year	Image IDs
Buea	2015	S1A_IW_GRDH_1SDV_20150412T173607_20150412T173632_005454_006F44_F045.SAFE<br>S1A_IW_GRDH_1SDV_20150611T173559_20150611T173624_006329_008515_3D8D.SAFE<br>S1A_IW_GRDH_1SDV_20150717T173601_20150717T173626_006854_0093D6_E3E2.SAFE<br>S1A_IW_GRDH_1SDV_20150822T173603_20150822T173628_007379_00A24B_E4D5.SAFE
	2020	S1A_IW_GRDH_1SDV_20200404T050603_20200404T050628_031973_03B14B_0153.SAFE<br>S1A_IW_GRDH_1SDV_20200404T050534_20200404T050603_031973_03B14B_3331.SAFE<br>S1A_IW_GRDH_1SDV_20200503T173644_20200503T173709_032404_03C085_879B.SAFE<br>S1A_IW_GRDH_1SDV_20200503T173619_20200503T173644_032404_03C085_2F42.SAFE<br>S1A_IW_GRDH_1SDV_20200603T050605_20200603T050630_032848_03CE03_75D9.SAFE<br>S1A_IW_GRDH_1SDV_20200603T050536_20200603T050605_032848_03CE03_409E.SAFE<br>S1A_IW_GRDH_1SDV_20200702T173648_20200702T173713_033279_03DB0E_7892.SAFE<br>S1A_IW_GRDH_1SDV_20200702T173623_20200702T173648_033279_03DB0E_839E.SAFE<br>S1A_IW_GRDH_1SDV_20200802T050609_20200802T050634_033723_03E899_324A.SAFE<br>S1A_IW_GRDH_1SDV_20200802T050540_20200802T050609_033723_03E899_EA67.SAFE
	2025	S1A_IW_GRDH_1SDV_20250402T050618_20250402T050643_058573_073FCA_7040.SAFE<br>S1A_IW_GRDH_1SDV_20250402T050549_20250402T050618_058573_073FCA_2B05.SAFE<br>S1A_IW_GRDH_1SDV_20250501T173700_20250501T173725_059004_075161_8792.SAFE<br>S1A_IW_GRDH_1SDV_20250501T173635_20250501T173700_059004_075161_2D20.SAFE<br>S1A_IW_GRDH_1SDV_20250601T050617_20250601T050642_059448_076127_7DE4.SAFE<br>S1A_IW_GRDH_1SDV_20250601T050548_20250601T050617_059448_076127_6B34.SAFE<br>S1A_IW_GRDH_1SDV_20250707T050615_20250707T050640_059973_07733C_E55A.SAFE<br>S1A_IW_GRDH_1SDV_20250707T050546_20250707T050615_059973_07733C_B86F.SAFE<br>S1A_IW_GRDH_1SDV_20250805T173655_20250805T173720_060404_078212_4A93.SAFE<br>S1A_IW_GRDH_1SDV_20250805T173630_20250805T173655_060404_078212_C3B1.SAFE
Kribi	2015	S1A_IW_GRDH_1SDV_20150412T173538_20150412T173607_005454_006F44_591A.SAFE<br>S1A_IW_GRDH_1SDV_20150611T173531_20150611T173559_006329_008515_68C8.SAFE<br>S1A_IW_GRDH_1SDV_20150712T050549_20150712T050618_006773_009181_47D1.SAFE<br>S1A_IW_GRDH_1SDV_20150817T050551_20150817T050620_007298_00A02A_2602.SAFE
	2025	S1A_IW_GRDH_1SDV_20250402T050618_20250402T050643_058573_073FCA_7040.SAFE<br>S1A_IW_GRDH_1SDV_20250501T173635_20250501T173700_059004_075161_2D20.SAFE<br>S1A_IW_GRDH_1SDV_20250501T173606_20250501T173635_059004_075161_DC22.SAFE<br>S1A_IW_GRDH_1SDV_20250601T050617_20250601T050642_059448_076127_7DE4.SAFE<br>S1A_IW_GRDH_1SDV_20250707T050615_20250707T050640_059973_07733C_E55A.SAFE<br>S1A_IW_GRDH_1SDV_20250805T173630_20250805T173655_060404_078212_C3B1.SAFE<br>S1A_IW_GRDH_1SDV_20250805T173601_20250805T173630_060404_078212_CAF7.SAFE
